# Supplementary material for: Antioxidant and Cytotoxic Properties of Berberis vulgaris (L.) Stem Bark Dry Extract
Source: Molecules. 2024 Apr 29;29(9):2053. doi: 10.3390/molecules29092053 (PMC11085362; doi:10.3390/molecules29092053)
Supplement: Supplementary file 1 [file molecules-29-02053-s001.zip › molecules-2965614-supplementary.pdf]

molecules

MDPI

Supplementary Material

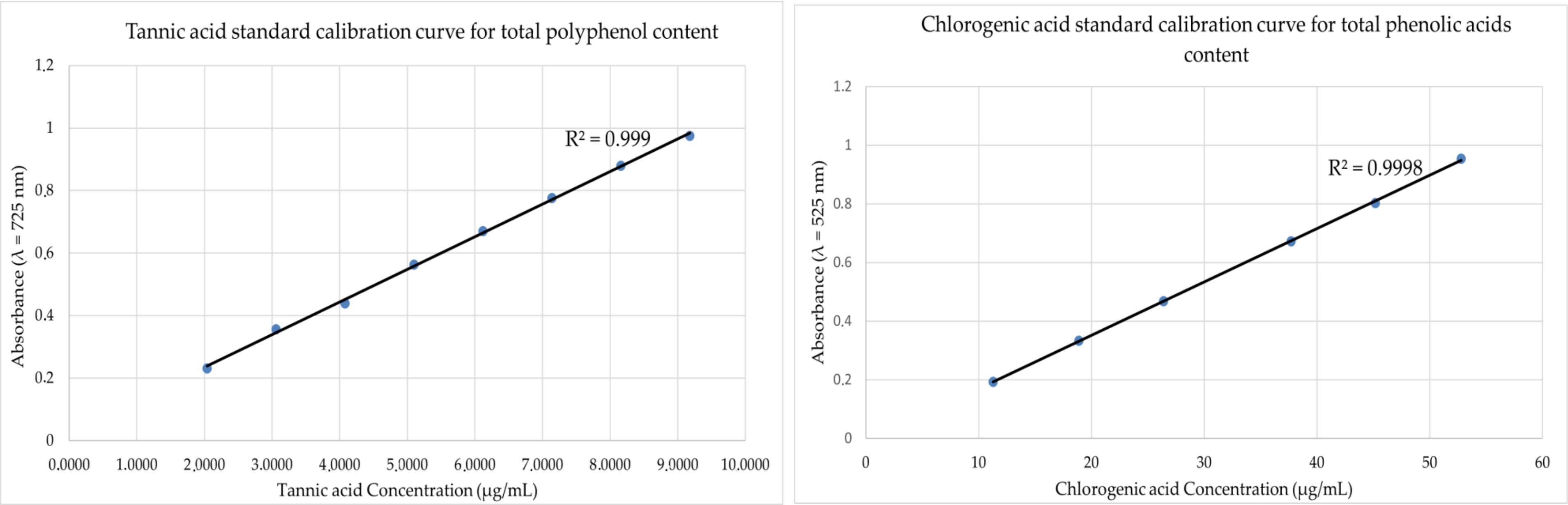

Figure S1. Standard calibration curves for TPC (left) and TPA (right).

Table S1. The antiproliferative effects of positive controls on normal cell and tumor cell lines after 24 and 48 hours of exposure.

**Exposure Time**	**24 h**			
		5-Fluorouracil		
**Cell line**	**HUVEC**	**LoVo**	**HT-29**	**HEP G2**
**[5-FU]**	*Cell viability % (mean ± SD)*			
3.125 µM	103.33 ± 7.05	94.26 ± 2.18	96.79 ± 3.59	98.04 ± 6.87
6.25 µM	102.78 ± 4.50	88.04 ± 1.45	92.31 ± 2.01	93.56 ± 0.52
12.5 µM	101.29 ± 2.46	79.31 ± 1.55	84.11 ± 2.69	84.10 ± 4.83
25 µM	103.74 ± 1.39	74.22 ± 3.82	80.87 ± 0.05	78.12 ± 3.61
50 µM	101.54 ± 7.05	62.41 ± 4.00	68.69 ± 4.01	66.85 ± 7.08
100 µM	102.60 ± 4.92	46.00 ± 2.91	54.74 ± 1.42	52.64 ± 3.15
200 µM	99.36 ± 3.36 [a,b,x]	34.27 ± 2.82 [a,c,y]	47.40 ± 4.59 [a,z]	41.69 ± 6.47 [b,c,w]
IC$_{50}$ (µM)	>200	<100	<200	<200
		Cisplatin		
**Cell line**	**HUVEC**	**SK-OV-3**	**PE/CA-PJ49**	
**[CisPt]**	*Cell viability % (mean ± SD)*			
3.125 µM	104.65 ± 4.15	99.76 ± 3.65	99.21 ± 1.17	
6.25 µM	102.12 ± 4.10	98.44 ± 0.78	97.87 ± 1.51	
12.5 µM	101.95 ± 3.69	95.24 ± 2.21	90.77 ± 0.82	
25 µM	102.35 ± 3.20	88.20 ± 3.82	86.01 ± 3.02	
50 µM	101.10 ± 2.21	79.43 ± 2.50	68.49 ± 4.98	
100 µM	100.04 ± 4.08	67.88 ± 0.95	44.95 ± 5.19	
200 µM	85.58 ± 4.62 [a,x]	52.61 ± 4.17 [a,y]	32.13 ± 4.12 [a,z]	
IC$_{50}$ (µM)	>200	>200	<100	
		Doxorubicin		
**Cell line**	**HUVEC**	**SK-OV-3**	**MDA-MB-231**	
**[DOX]**	*Cell viability % (mean ± SD)*			
0.625 µM	103.67 ± 1.97	98.66 ± 1.91	90.65 ± 2.20	
1.25 µM	101.56 ± 2.79	96.06 ± 1.07	77.14 ± 4.67	
2.5 µM	102.59 ± 3.20	93.55 ± 1.67	72.99 ± 0.92	
5 µM	100.49 ± 5.24	89.00 ± 0.36	62.86 ± 4.78	
10 µM	101.74 ± 1.31	82.22 ± 1.91	55.71 ± 1.65	
20 µM	100.64 ± 0.41	71.81 ± 2.62	44.81 ± 3.02	
40 µM	98.87 ± 5.49 [a,x]	61.59 ± 1.85 [a,y]	33.38 ± 6.90 [a,z]	

IC_{50} (μM)	>40	>40	>40

**Exposure Time**	**48 h**			
	5-Fluorouracil			
**Cell line**	**HUVEC**	**LoVo**	**HT-29**	**HEP G2**
**[5-FU]**	*Cell viability% (mean ± SD)*			
3.125 μM	105.50 ± 1.72	90.30 ± 4.50	93.08 ± 4.25	96.77 ± 5.75
6.25 μM	103.63 ± 2.04	82.36 ± 2.19	82.16 ± 4.62	90.00 ± 1.79
12.5 μM	102.48 ± 4.96	66.02 ± 5.61	77.37 ± 1.70	81.00 ± 3.47
25 μM	99.02 ± 3.36	59.92 ± 3.98	71.50 ± 5.12	69.12 ± 0.27
50 μM	96.51 ± 2.42	49.30 ± 2.93	55.21 ± 1.25	56.93 ± 4.15
100 μM	93.99 ± 2.29	31.94 ± 3.57	42.78 ± 1.76	46.97 ± 5.44
200 μM	88.63 ± 4.52 ^{a,b,x}	13.25 ± 3.49 ^{a,b,y}	28.24 ± 7.59 ^{a,z}	25.50 ± 6.68 ^{b,w}
IC_{50} (μM)	>200	<50	<100	<100

	Cisplatin		
**Cell line**	**HUVEC**	**SK-OV-3**	**PE/CA-PJ49**
**[CisPt]**	*Cell viability % (mean ± SD)*		
3.125 μM	101.2 ± 2.35	94.34 ± 4.56	97.08 ± 3.45
6.25 μM	100.68 ± 1.46	82.70 ± 1.88	90.37 ± 4.75
12.5 μM	96.43 ± 5.70	75.19 ± 0.27	84.28 ± 1.60
25 μM	86.59 ± 0.38	67.87 ± 1.72	73.16 ± 4.26
50 μM	78.02 ± 3.12	55.38 ± 6.05	60.26 ± 6.52
100 μM	68.79 ± 1.02	34.32 ± 3.97	33.06 ± 5.19
200 μM	55.80 ± 1.97 ^{b,c,x}	29.77 ± 2.04 ^{b,y}	21.18 ± 6.53 ^{c,z}
IC_{50} (μM)	>200	<100	<100

	Doxorubicin		
**Cell line**	**HUVEC**	**SK-OV-3**	**MDA-MB-231**
**[DOX]**	*Cell viability % (mean ± SD)*		
0.625 μM	101.08 ± 0.57	88.91 ± 2.58	85.04 ± 0.95
1.25 μM	103.29 ± 5.41	81.30 ± 2.63	74.76 ± 0.51
2.5 μM	101.15 ± 4.07	77.86 ± 1.13	68.99 ± 3.58
5 μM	100.26 ± 1.78	71.86 ± 4.35	53.84 ± 6.21
10 μM	94.72 ± 0.95	62.91 ± 1.18	46.51 ± 4.30
20 μM	91.97 ± 2.04	55.54 ± 1.07	37.81 ± 6.33
40 μM	90.02 ± 0.00 ^{b,x}	36.42 ± 3.43 ^{b,y}	19.21 ± 1.91 ^{b,z}

IC_{50} (µM)	>40	<40	<10

HUVEC — human umbilical endothelial cells; HEP G2 — human hepatocellular carcinoma; HT-29 and LoVo — human colon adenocarcinomas; MDA-MB-231 —human breast adenocarcinoma; PE/CA-PJ49 — human squamous tongue carcinoma; SK-OV-3 — human ovary adenocarcinoma; SD—standard deviation. The superscript letters indicate the significant statistical differences ($p < 0.05$): [a, b, c, d] in the same column, between rows; [x,y,z] in the same row, between columns. Interpretation of IC_{50} values is based on [16]: $IC_{50} \leq 10$ µM = good cytotoxicity, 10 µM < $IC_{50} \leq 30$ µM = low cytotoxicity; $IC_{50} > 30$ µM = inactive. Data shown are expressed as mean values ± standard deviations (SD) of three different experiments ($n = 3$)

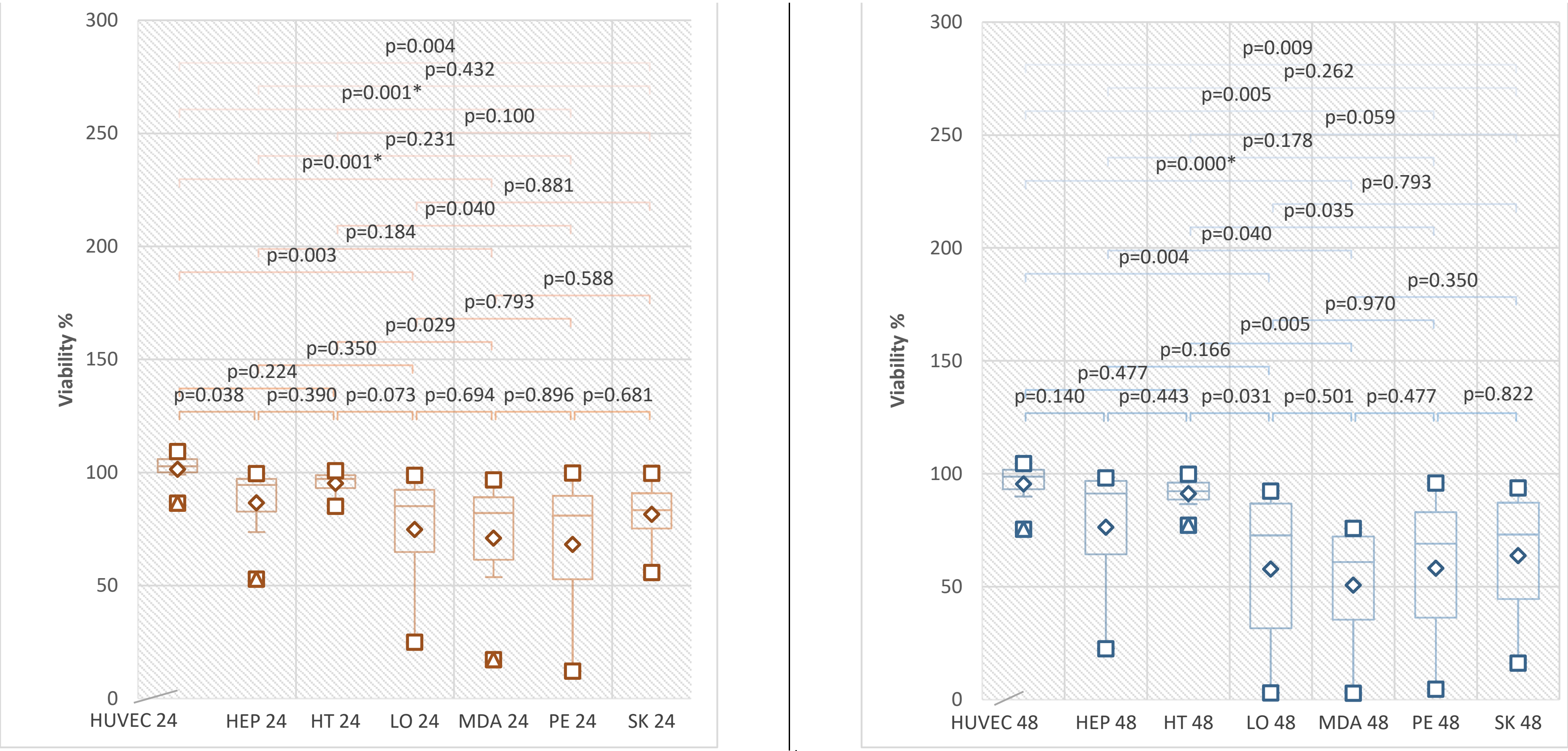

(a) **(b)**

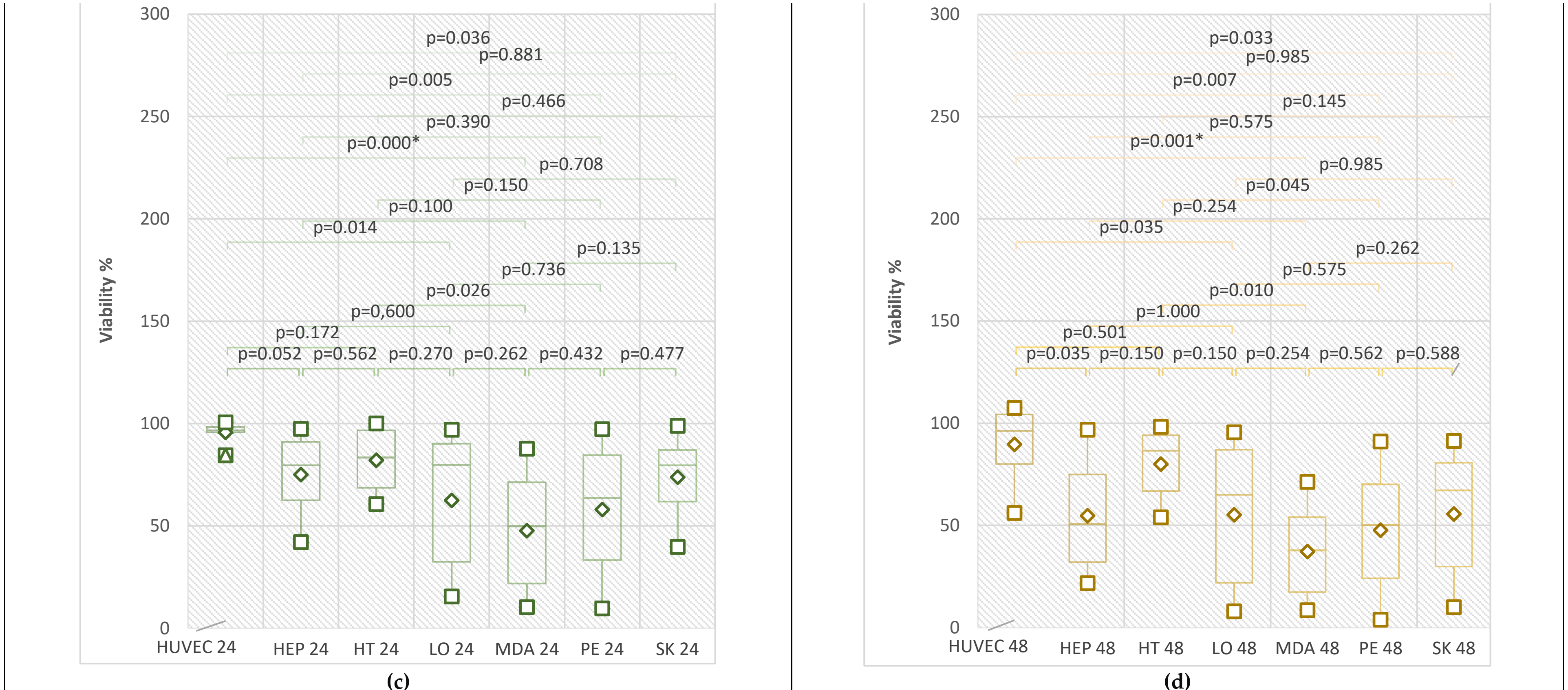

(c) **(d)**

Figure S2. The viability% (V%) of normal and tumor cells exposed to BVE (a,b) and BS (c,d) various concentrations for 24 h (a,c) and 48 h (b,d). Bonferroni-corrected significance level at $\alpha = 0.05$ is 0,0024; *p-value < 0.024 corresponds to α < 0,05 and shows statistically significant differences. HUVEC — human umbilical endothelial cell; HUV—HUVEC — human umbilical endothelial cell; HEP—HEP G2 — human hepatocellular carcinoma; HT—HT-29 and LO—LoVo — human colon adenocarcinomas; MDA—MDA-MB-231 —human breast adenocarcinoma; PE—PE/CA-PJ49 — human squamous tongue carcinoma; SK—SK-OV-3 — human ovary adenocarcinoma; 24 and 48 —period of exposure (hours); c — control (BS); BVE—*B. vulgaris* dry hydro-ethanol extract; BS—Berberine standard.

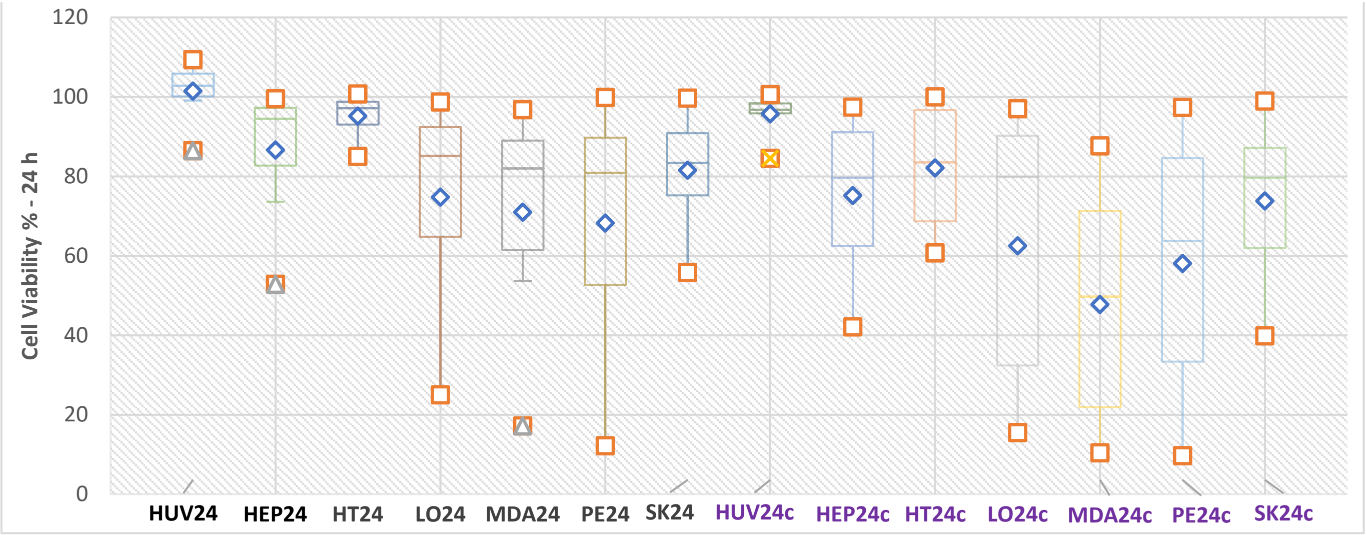

(a)

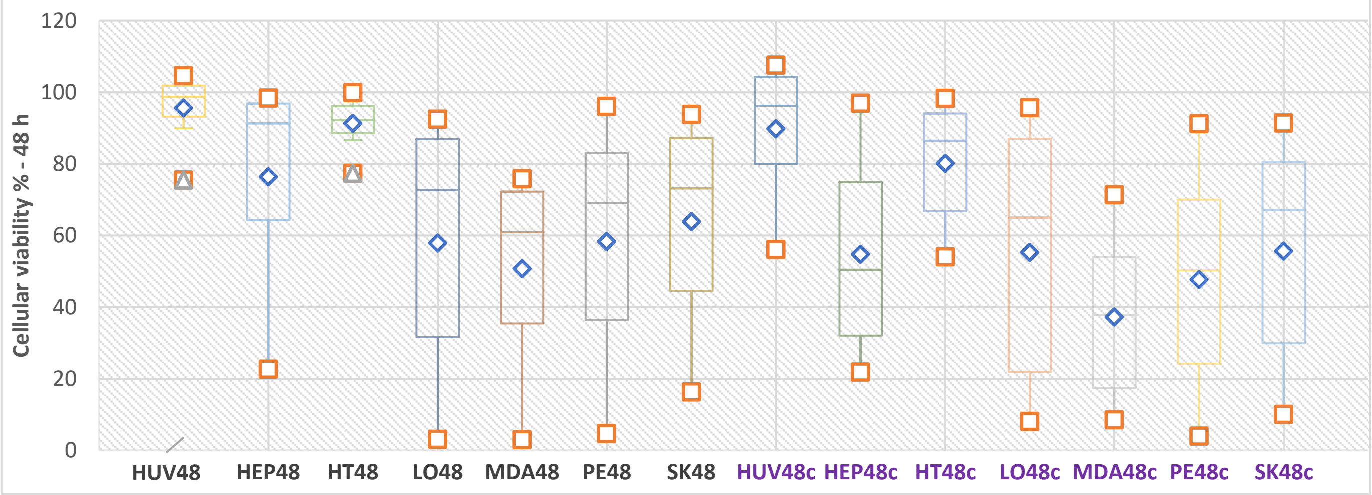

(b)

Figure S3. Comparison between BVE and BS cytotoxicity (quantified as cell viability %) after 24 h (a) and 48 h (b) of contact. HUV—HUVEC — human umbilical endothelial cell; HEP—HEP G2 — human hepatocellular carcinoma; HT—HT-29 and LO—LoVo — human colon adenocarcinomas; MDA—MDA-MB-231 —human breast adenocarcinoma; PE—PE/CA-PJ49 — human squamous tongue carcinoma; SK—SK-OV-3 — human ovary adenocarcinoma; 24 and 48 —period of exposure (hours); c — control (BS); BVE—*B. vulgaris* dry hydro-ethanol extract; BS—Berberine standard

Table S2. Pearson correlation between the cytotoxic effects of BVE, BS, and anticancer drugs (expressed as cell viability %) after 24 and 48 hours of treatment on tested cell lines

Variables	BVE24	BS24	CisPt24	5-FU24	DOX24	BVE48	BS48	CisPt48	5-FU48	DOX48
BVE24	**1**									
BS24	**0.906**	**1**								
CisPt24	0.469	0.644	**1**							
5-FU24	0.619	**0.813**	0.414	**1**						
DOX24	0.573	0.747	**0.934**	0.482	**1**					
BVE48	**0.910**	**0.935**	0.545	**0.866**	0.597	**1**				
BS48	**0.866**	**0.928**	0.522	**0.853**	0.595	**0.955**	**1**			
CisPt48	0.471	0.649	**0.997**	0.454	**0.918**	0.572	0.543	**1**		
5-FU48	0.587	0.624	0.428	**0.782**	0.500	**0.788**	0.715	0.469	**1**	
DOX48	0.523	0.609	**0.837**	0.405	**0.920**	0.552	0.520	**0.830**	0.667	**1**

Values in bold show are statistically significant ($p<0.05$). BVE — dry hydro-ethanolic extract of *Berberis vulgaris* cortex; BS — berberine sulfate hydrate; CisPt — Cisplatin; DOX — Doxorubicin, 5-FU —5-Fluorouracil; 24 and 48 — treatment time (24 and 48 hours).

Table S3. Pearson correlation between BVE secondary metabolites content, antioxidant activity, and cytotoxicity expressed as cell viability % after 24 and 48 hours of treatment.

Variables	HUVEC 24	HUVEC 48	HEP 24	HEP 48	HT 24	HT 48	LO 24	LO 48	MDA 24	MDA 48	PE 24	PE 48	SK 24	SK 48	TPC	TPA	DPPH	ABTS	FRAP
HUVEC 24	**1**	**0.970**	0.941	0.878	0.929	**0.968**	0.926	0.746	0.942	0.872	0.894	0.818	**0.951**	0,814	-0,914	-0,904	-0,945	-0,942	-0,949
HUVEC 48	**0.970**	**1**	**0.989**	**0.959**	**0.991**	**1.000**	**0.988**	0.882	**0.995**	**0.965**	**0.976**	0.932	**0.997**	0,926	**-0,979**	**-0,980**	**-0,996**	**-0,993**	**-0,995**
HEP 24	0.941	**0.989**	**1**	**0.988**	**0.994**	**0.988**	**0.996**	0.924	**0.985**	**0.978**	**0.978**	**0.959**	**0.983**	**0,961**	**-0,997**	**-0,979**	**-0,990**	**-0,976**	**-0,979**
HEP 48	0.878	**0.959**	**0.988**	**1**	**0.986**	**0.959**	**0.990**	**0.969**	**0.967**	**0.988**	**0.978**	**0.986**	**0.960**	**0,990**	**-0,997**	**-0,976**	**-0,973**	**-0,954**	**-0,955**
HT 24	0.929	**0.991**	**0.994**	**0.986**	**1**	**0.991**	**0.999**	0.938	**0.996**	**0.991**	**0.994**	**0.972**	**0.994**	**0,968**	**-0,995**	**-0,995**	**-0,998**	**-0,991**	**-0,991**
HT 48	**0.968**	**1.000**	**0.988**	**0.959**	**0.991**	**1**	**0.989**	0.884	**0.996**	**0.966**	**0.978**	0.933	**0.998**	0,927	**-0,979**	**-0,982**	**-0,997**	**-0,994**	**-0,996**
LO 24	0.926	**0.988**	**0.996**	**0.990**	**0.999**	**0.989**	**1**	0.942	**0.993**	**0.991**	**0.992**	**0.974**	**0.990**	**0,972**	**-0,998**	**-0,992**	**-0,996**	**-0,986**	**-0,987**
LO 48	0.746	0.882	0.924	**0.969**	0.938	0.884	0.942	**1**	0.913	**0.973**	**0.955**	**0.993**	0.900	**0,994**	-0,949	-0,947	-0,917	-0,901	-0,896
MDA 24	0.942	**0.995**	**0.985**	**0.967**	**0.996**	**0.996**	**0.993**	0.913	**1**	**0.982**	**0.992**	**0.956**	**1.000**	0,947	**-0,982**	**-0,995**	**-0,999**	**-0,999**	**-0,999**
MDA 48	0.872	**0.965**	**0.978**	**0.988**	**0.991**	**0.966**	**0.991**	**0.973**	**0.982**	**1**	**0.997**	**0.994**	**0.976**	**0,989**	**-0,988**	**-0,995**	**-0,983**	**-0,976**	**-0,974**
PE 24	0.894	**0.976**	**0.978**	**0.978**	**0.994**	**0.978**	**0.992**	**0.955**	**0.992**	**0.997**	**1**	**0.983**	**0.988**	**0,975**	**-0,984**	**-1,000**	**-0,991**	**-0,989**	**-0,987**
PE 48	0.818	0.932	**0.959**	**0.986**	**0.972**	0.933	**0.974**	**0.993**	**0.956**	**0.994**	**0.983**	**1**	0.946	**0,998**	**-0,976**	**-0,979**	**-0,958**	-0,947	-0,943
SK 24	**0.951**	**0.997**	**0.983**	**0.960**	**0.994**	**0.998**	**0.990**	0.900	**1.000**	**0.976**	**0.988**	0.946	**1**	0,937	**-0,978**	**-0,991**	**-0,999**	**-0,999**	**-1,000**
SK 48	0.814	0.926	**0.961**	**0.990**	**0.968**	0.927	**0.972**	**0.994**	0.947	**0.989**	**0.975**	**0.998**	0.937	**1**	**-0,978**	**-0,970**	**-0,951**	-0,935	-0,932
TPC	-0.914	**-0.979**	**-0.997**	**-0.997**	**-0.995**	**-0.979**	**-0.998**	-0.949	**-0.982**	**-0.988**	**-0.984**	**-0.976**	**-0.978**	**-0,978**	**1**	**0,983**	**0,987**	**0,972**	**0,973**
TPA	-0.904	**-0.980**	**-0.979**	**-0.976**	**-0.995**	**-0.982**	**-0.992**	-0.947	**-0.995**	**-0.995**	**-1.000**	**-0.979**	**-0.991**	**-0,970**	**0,983**	**1**	**0,994**	**0,993**	**0,991**
DPPH	-0.945	**-0.996**	**-0.990**	**-0.973**	**-0.998**	**-0.997**	**-0.996**	-0.917	**-0.999**	**-0.983**	**-0.991**	**-0.958**	**-0.999**	**-0,951**	**0,987**	**0,994**	**1**	**0,997**	**0,998**
ABTS	-0.942	**-0.993**	**-0.976**	**-0.954**	**-0.991**	**-0.994**	**-0.986**	-0.901	**-0.999**	**-0.976**	**-0.989**	-0.947	**-0.999**	-0,935	**0,972**	**0,993**	**0,997**	**1**	**1,000**
FRAP	-0.949	**-0.995**	**-0.979**	**-0.955**	**-0.991**	**-0.996**	**-0.987**	-0.896	**-0.999**	**-0.974**	**-0.987**	-0.943	**-1.000**	-0,932	**0,973**	**0,991**	**0,998**	**1,000**	**1**

Values in bold are different from 0 with a significance level alpha=0,05

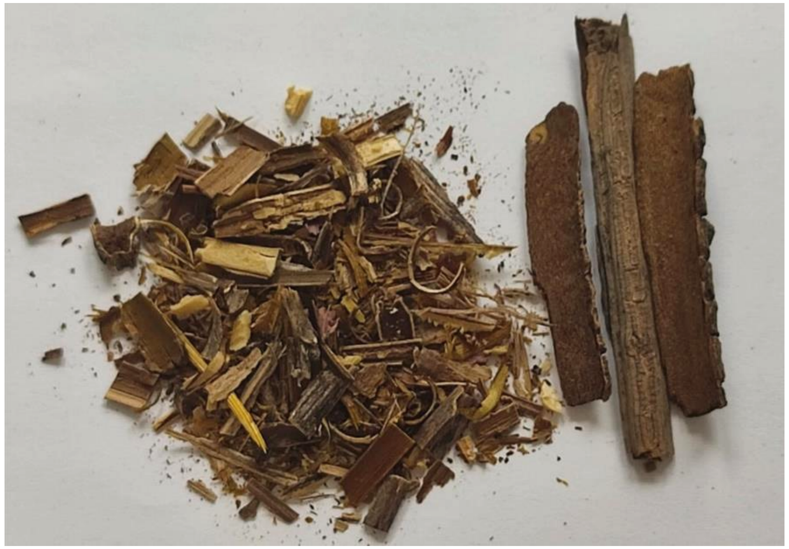

Figure S4. Berberidis cortex.
